# Comparison between two different algorithms used for pretreatment QA via aSi portal images

**DOI:** 10.1120/jacmp.v16i3.5202

**Published:** 2015-05-08

**Authors:** Charbel Merheb, Clément Chevillard, Wassim Ksouri, Maher Fawzi, Marc Bollet, Alain Toledano

**Affiliations:** ^1^ Department of Physics Hartmann Institute Levallois‐Perret France; ^2^ Department of Physics Joseph Fourier University Saint‐Martin‐d'Heres France

**Keywords:** EPID, pretreatment QA, IMRT, VMAT, amorphous silicon, GLAaS, Varian portal dosimetry

## Abstract

Several algorithms exist to perform quality assurance for volumetric‐modulated arc therapy (VMAT) treatments based on electronic portal imaging devices (EPID). These algorithms are used to compare doses (convert into water, GLAaS) and fluences (in amorphous silicon (aSi), Varian portal dosimetry). The aim of this study is to compare the two methods using clinical data. In this study, Varian portal dosimetry (VPD) and Epiqa solutions were compared. We used a same set of patient images data treated with 6 MV and 20 MV photon energies and different locations. The response of the portal imaging device was also investigated with different field sizes, monitor units, dose rates, sag effect, and linac daily output. All images were acquired on an electronic portal imaging device (EPID) positioned at source detector distance (SDD) of 100 cm. A virtual water phantom was used for Epiqa to calculate the dose matrices at the maximum depth doses $d_{\text{max}}$. The 2D gamma evaluation index (GAI) was performed to quantitatively compare the results given by the two solutions. The response of the EPID gave a good agreement with Epiqa (deviation less than 1%) for MU greater than 20 for both 6 MV and 20 MV photon energies. For VPD, the upward sloping trend showed a good agreement for MU higher than 50. Dose rate evaluations for both methods gave a deviation of, respectively, 0.4 and 0.5 % for 6 MV and 20 MV. The gamma criteria of 3 mm for distance to agreement and 3 % for dose difference was, as mean ±1 SD, 99.81%±1.48% and 99.42%±0.97% for VPD and Epiqa, respectively, for 6 MV photon energy. The mean values of the gamma criteria for the collected data using 20 MV photon energy were, respectively, 98.33%±2.41% and 98.12%±1.99% for VPD and Epiqa. The output constancy deviation correction (a $10\times 10\;{\text{cm}}^{2}$ reference field plan to obtain absorbed dose despite the linac monitor daily variations) showed a mean deviation of, respectively, 0.07%±0.57% and 0.16%±1.38% for 6 MV and 20 MV photon energies. For sag effect, a slight improvement was noticed for realignment of the integrated image and was 0.25%±0.69% for 6 MV and 0.40%±0.57% for 20 MV. The clinical data were used for pretreatment QA with the two systems, both VPD and Epiqa software, showed acceptable and similar results for low and high energies. Furthermore, Epiqa shows better linearity response for low MU.

PACS number: 87.53.Bn, 87.55.km, 87.57.uq

## I. INTRODUCTION

RapidArc is a technique for volumetric delivery of intensity‐modulated arcs, based on the concept published by Otto.^(1)^ This dynamic technique achieves an entire intensity‐modulated treatment in a single gantry rotation around the patient.^(2)^ However, the complexity of treatment delivery and modulated dose to the target volume requires a pretreatment quality assurance (QA) for each delivered arc of patient treatment.^3^, ^4^, ^5^ Electronic portal imaging device (EPID) plays a key role in the chain of verification procedures (QA program) in external beam radiotherapy. It provides a safety for both simple and advanced treatments. It is the preferred device for patient positioning and dose verification in radiotherapy. The interest in EPID dosimetry is due to its favorable characteristics, such as ease of use, linear response to radiation, fast image acquisition, high resolution, and digital format.^(6)^

This system uses amorphous silicon (aSi) detector exposed to the photon beam. Measurements are realized in a 2D matrix, perpendicular to the beam axis.

QA can follow two distinct approaches:

1. The fluences from the treatment planning systems (TPS) are directly compared to the fluence matrix calculated from detector reading knowing the MLC movement and instantaneous output in aSi. This is what the Varian portal dose image prediction (VPD) software does using calibration units (CU) to compare the calculated and measured fluencies.
2. The aSi detector reading is converted to a dose at the maximum depth dose in water then compared to estimated dose calculated in water, and then by the TPS.^7^, ^8^

This work describes our investigation in order to compare the two approaches described above. Firstly, we compared the accuracy of these two approaches (Approach 1: VPD directly in aSi, and Approach 2: EPIQA calculated in virtual water phantom) according to delivered monitor units, field size, and dose rate, using a static field with simple open beams. Then, we compared the results given by these two methods for patients' pretreatment QA. Both investigations were realized with 6 and 20 MV photon energies.

## II. MATERIALS AND METHODS

### A. The portal vision imager

In this study, images were acquired with the IAS3 electronic imaging device (Varian Medical Systems, Palo Alto, CA). This system is coupled to a Varian 2300iX linac accelerator with 6 and 20 MV photon energies and 120‐leaf Varian Millennium multileaf collimator (MLC) via the PortalVision Exact‐Arm (Varian Medical Systems).^(9)^ The device contains a Portal Vision imager (PV) aS1000 and consists of a principal plate with a total thickness of 8 mm, a thin slice of 1 mm of copper, a film of phosphor (0.5 mm of Gd2O2S:Tb Kodak Lanex Fast B), and a 2D matrix detection.^(10)^ This one is made of a thin slice of amorphous silicon with above a matrix of photodiodes and transistors with field effect (thin‐film transistors, TFTs). The detection matrix contains 1024×768 pixels distributed on a $40\times 30\;{\text{cm}}^{2}$ active area, with a high resolution (0.392 mm).^(11)^

### B. Calibration of the EPID system

In order to use this MV imaging system for dosimetry, we followed Varian specifications to calibrate the system.

#### B.1 Dark field correction

Dark field image is acquired without radiation. Its contents reflect array imperfections.

#### B.2 Flood field (FF) correction

FF image represents the field homogeneity and individual cell sensitivities. The FF calibration then corrects the signals generated from the cassettes, assuming that the treatment beam has uniform intensity. The sensitivity of each pixel is determined periodically by acquiring an image with radiation and a wide open field. For SDD of 100 cm, the data are acquired with a $40\times 30\;{\text{cm}}^{2}$ field size for both 6 MV and 20 MV and different dose rates (100 MU/min to 600 MU/min).

#### B.3 Beam profile correction

A diagonal profile of the largest field size at 8 mm depth is provided. The software generates a radially symmetric correction based on the entered profile.

#### B.4 Dose normalization

The signals generated by the radiation beam are related to dose. When the calibration is performed, radiation beam is related to calibration units (CU). The calibration is performed with a $10\times 10\;{\text{cm}}^{2}$ open field and 100 MU. EPID response was scaled so that 1 CU corresponds to 1 MU delivered.

The dark and flood field corrections are regularly performed in order to record pixel offset and background noise obtained in the absence of radiation. Dosimetric calibration of the imager, including dark and flood field corrections and a whole calibration measurement, is performed as well for 6 MV and 20 MV photon energies available in our center.

### C. The portal dosimetry prediction algorithm

The VPD algorithm, portal dose image prediction (PDIP) version 10.0, can be used for both IMRT and VMAT techniques. For these arcs, the TPS (Eclipse, Varian Medical Systems) calculates the planned fluence by summing up the individual aperture fluencies from the control points of the field. Each control points defining multileaf collimator (MLC) shape, MLC segment dose, and gantry‐angle window. The dynamic dose rate is also accounted for. The PDIP algorithm calculates the predicted EPID image based on this sum fluence in the same way as for IMRT fields. The distribution is defined at isocenter and has a fixed resolution of $2.5\times 2.5\;{\text{mm}}^{2}$.^(12)^ The portal dose image is calculated by convolving the fluence with Gaussian kernels, as in Eq. (1):

(1) $$P=f^{\prime }⊗k\cdot {\left(\frac{SAD}{SDD}\right)}^{2}\cdot \left(\frac{OF(x,y)}{PSF(x,y)}\right)$$

where *P* is the calculated portal dose image in terms of CU; $f^{\prime }$ is the input fluence corrected by the intensity profile and scaled by detector distance; ⊗ is the convolution operator; *k* represent the portal imager dose kernel; *SDD* is the source‐to‐detector distance of the portal image measurement; *SAD* is the source‐to‐axis distance of the treatment unit; *x* represents the field size at the SAD in the X direction; *y* represents the field size at the SAD in the Y direction; PSF(x,y) is the phantom scatter factor for field size ${\text{fs}}_{x},{\text{fs}}_{y}$, defined at SAD; and OF(x,y) is the output factor for field size ${\text{fs}}_{x},{\text{fs}}_{y}$, defined at SAD and normalized to a $10\times 10\;{\text{cm}}^{2}$ field.

The analytical function describing the kernel as a function of radial distance r from the pencil beam is modeled as a sum of three Gaussian contributions. Thus, the model does not take into account the arm backscatter effect.^(13)^

### D. Epiqa solution based on the GLAaS algorithm

The algorithm used by the commercial software Epiqa version 2.1.2 from EPIdos s.r.o. (Bratislava, Slovakia) has been described in numerous previous articles by Nicolini et al.^(7)^ We are given in the following a brief overview of how it works.

The GLAaS algorithm is used to convert an image acquired with EPID into a matrix dose distribution at a certain depth in water. For a given beam, the response of the aSi in the detectors is linear:

(2) D(Gy)=m×R+q

where *D* is the dose in Gy measured with an ion chamber; *m* and *q* are the slope and the intercept for a field of size equivalent window width field; and *R* is the total PV reading.

During an IMRT treatment, the field's shape is continuously changing. GLAaS accounts for those changes in times and position, using different m and q values, and takes into account primary and transmitted radiation on pixel by pixels basis.

The total dose $d_{i}$ in the ith pixel over the entire IMRT field delivery is:

(3) $$d_{i}=d_{pr,i}+d_{tr,i}$$

where *pr* and *tr* indices are, respectively, used for primary and transmitted radiation.

Epiqa was configured using the so‐called “mix‐setup”. Measured PV images are acquired without adding any buildup on the top of the cassette; therefore, the measurement depth is 0.8 cm, which is the intrinsic water‐equivalent thickness of the EPID device, but matrices are converted into dose at $d_{\text{max}}$. Dose‐converted measurements are then compared with the dose computed by the TPS at $d_{\text{max}}$.^7^, ^8^

For each treatment planning, a verification plan was created in a virtual water phantom perpendicular to the beam axis using the original parameters (MLC and dose rate). An image of a $10\times 10\;{\text{cm}}^{2}$ field with 50 MU is acquired before each set of measurements to obtain absorbed dose despite the linac monitor daily variations. The verification plan was delivered on a fixed gantry angle of 0°. The used dose calculation algorithm was AAA; for the current study we choose 1 mm as grid size, being the finest allowed resolution. For the QA verification with Epiqa, the size of the grid is the same as for patient plan dose calculation (2.5 mm). The planar dose at $d_{\text{max}}$ is then 98.5 cm and 96.5 cm for 6 MV and 20 MV photon energies, respectively. Plan dose is then exported with a matrix of 512×512 size. The dose matrix computed by Epiqa is convolved with a Gaussian distribution with a 1 mm sigma value.

### E. Evaluation tools

The gamma agreement index (GAI), defined as the percentage of point inside the field passing the gamma criteria, was computed for each patient. Evaluation was performed into a zone defined as the area with dose higher than 5% of the maximum dose. Threshold criteria were chosen as following: ΔD (dose difference)/DTA (distance to agreement) of 3%/3 mm.^(14)^ The 2%/2 mm and 4%/4 mm criteria were also used for the comparison. Calculated image was considered as reference for the two approaches. Percentage dose values are normalized to the maximum dose in the plane. For Epiqa, ΔD% is referred to the maximum significant dose per field of the reference image, test image or any value defined by user. Maximum significant dose is defined as the maximum dose value in the histogram of pixel values in the field minus the highest 5% (default) pixel values in the histogram. This option, introduced to avoid bias in the analysis due to presence of high dose peaks in the measured data, was not used since VPD does not permit such kind of analysis. The consideration of EPID sag during arc treatments could be corrected by VPD tools by a realignment of the integrated image in order to enhance dosimetry results. Since EPID sag correction is not available with Epiqa, we did not use this option with VPD. Nevertheless, an evaluation of this effect was assessed by comparing corrected and noncorrected results for VPD in term of gamma criteria. Correction was performed with the automatic alignment algorithm. Linac monitor daily variation could be taken into account by Epiqa. VPD does not permit such correction, thus this option was not used into Epiqa for comparison with VPD. However, the impact of this correction was assessed for the same set of images.

### F. Detector response

To determine the accuracy of VPD and Epiqa solutions, the response of the detector with the two solutions in function of MU, field sizes, and dose rate was investigated.^(11)^ Measurements were compared with the dose maps given by the TPS. For MU linearity response, acquisitions of a $10\times 10\;{\text{cm}}^{2}$ open field were realized with 5, 10, 20, 50, 100, 300, and 500 MU. The dose rate was fixed to 600 MU/min.

The evaluation of the field size dependence was performed for 3, 5, 7, 10, 15, 20, and 28 cm square field sizes. For each field, we delivered 100 MU and 600 MU/min dose rate.

The dose rate linearity response was checked using a $10\times 10\;{\text{cm}}^{2}$ open field with different dose rates of 100, 200, 300, 400, 500 and 600 MU/min. Cumulated dose for each field was fixed to 100 MU.

The position of the EPID during measurements was fixed to a SDD of 100 cm.

For these static fields, the evaluation was performed into the area with values higher than 90% of the maximum. A mean dose is then calculated with a standard deviation. In order to get a complementary accurate evaluation, measurements were also corrected from the output constancy deviation of the machine in order to identify the different problems. The latter was measured before the set of acquired images.

### G. VMAT patient QA acquisitions

A total of 77 (38 for 6 MV and 39 for 20 MV) patients were included in this study with 100 plans and 200 arcs for both 6 MV and 20 MV photon energies (100 arcs per energy). Patients were randomly chosen over all VMAT treatments over 2 years. 6 MV correspond to head and neck, while prostate and pelvis were the principal indications for 20 MV treatments. Measurements of the QA were performed after having verified each individual arc onto the treatment machine. Results of the pretreatment QA were summarized in terms of gamma criteria.

## III. RESULTS

### A. Open field analysis

**Figure 1:**
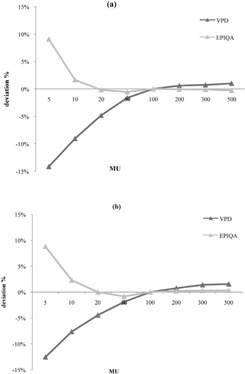
Difference between measured and calculated dose, function of monitored unit for photons 6 MV (a) and 20 MV (b) photon energies.

The three results are shown in 1, 2, 3. MU analysis is shown in Fig. 1. When taking into account MU, Epiqa showed a linear response for irradiations above 20 MU (deviation less than 0.2% for the two photon energies). Below 20 MU, Epiqa overestimates the dose response, but remains acceptable between 10 and 20 MU (1.7% for low energy and 2.3% for high energy). The VPD algorithm showed worse responses for low MU; in fact, for 50 MU, the error is about −1.5% for 6 MV and −1.9% for 20 MV.

**Figure 2:**
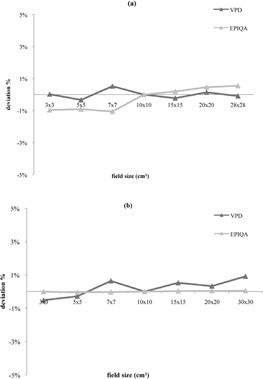
Difference between measured and calculated dose, function of field size for 6 MV (a) and 20 MV (b) photon energies.

**Figure 3:**
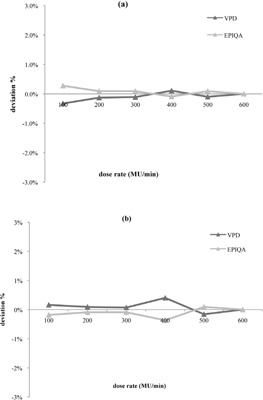
Difference between measured and calculated dose, function of dose rate for 6 MV (a) and 20 MV (b) photon energies.

The agreement for the set of field size measurements between the two algorithms was less than 1% for 6 MV and 20 MV photon energies for all field sizes (Fig. 2). However Epiqa‐measured data show a slight raise of the response in function of field size for 6 MV compared to VPD at the same energy. Conversely, the deviations from the predicted value rise with VPD.

The deviation between measured and calculated image data show independent variation in function of dose rate for both energies. In fact, the maximum deviation was less than 0.3% for 6 MV (Fig. 3(a)) and less than 0.4% for 20 MV (Fig. 3(b)).

### B. VMAT patient analysis

**Figure 4:**
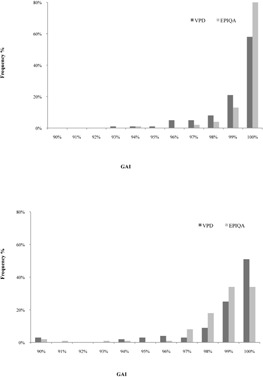
Gamma criteria results (ΔD=3%,DTA=3 mm) for all acquisitions.

Table 1 summarizes gamma criteria results for both calculation methods and 6 MV and 20 MV photon energies. For low energy, the mean value of VPD gamma criteria was 98.8%±1.5% and 99.4%±1% for Epiqa using the clinical criteria to score the gamma criteria. For high photon energy we obtained 98.3%±2.4% and 98.1%±2% for VPD and Epiqa, respectively, and the previous criteria. The presented histograms in Fig. 4 illustrate the results in terms of gamma criteria values obtain for each QA patient. For low energy, 93% of the total fields gave a gamma criteria score over 99% using Epiqa vs. 79% using VPD. The histograms related to high‐energy (20 MV) results present left‐skewed and similar shapes for both algorithms and decrease slightly more in term of gamma criteria compared to low‐energy (6 MV) histograms.

**Table 1:** Summary of VPD and Epiqa results: GAI (gamma criteria) values for different threshold criteria.

			*GAI (%)*	*GAI (%)*	*GAI (%)*
*Energy*	*Calculation Tool*		[ΔD/DTA=3%/3 mm]	[ΔD/DTA=2%/2 mm]	[ΔD/DTA=4%/4 mm]
6 MV	VPD	Mean	98.81±1.48	94.28±4.52	99.65±0.70
		95% confidence level	[98.5, 99.1]	[93.4, 95.2]	[99.5, 99.8]
	Epiqa	Mean	99.42±0.97	92.11±5.53	99.96±0.15
		95% confidence level	[99.2, 99.6]	[91.0, 93.2]	[99.9, 100.0]
20 MV	VPD	Mean	98.33±2.41	90.64±6.18	99.56±1.13
		95% confidence level	[97.9, 98.8]	[89.4, 91.8]	[99.3, 99.8]
	Epiqa	Mean	98.12±1.99	90.18±4.54	99.54±1.07
		95% confidence level	[97.7, 98.5]	[89.3, 91.1]	[99.3, 99.7]

**Figure 5:**
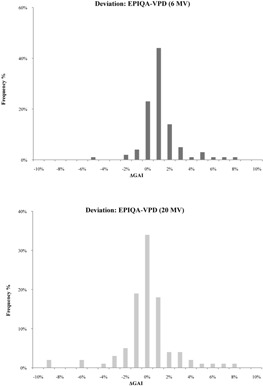
Results of ΔGAI (gamma criteria) for Epiqa and VPD, respectively, 6 and 20 MV photon energies and [ΔD/DTA=3%/3 mm].

The difference of GAI between Epiqa and VPD are presented in Table 2 for 6 MV and 20 MV photon energies. For the former, we obtained a deviation of 0.6%±1.7% between Epiqa and VPD. For the latter, a deviation between both calculation methods was −0.21%±2.94%. A negative value means that VPD gives a better result than Epiqa. Figure 5 shows the difference between Epiqa and VPD in term of gamma criteria for ΔD/DTA of 3%/3 mm. We notice that the shapes of the two curves follow a standard normal distribution. For 20 MV photon energy, the curve tends to be more spread out.

**Table 2:** Results of ΔGAI of Epiqa and VPD respectively, 6 and 20 MV photon energies for different threshold criteria.

		ΔGAI(%)	ΔGAI(%)	ΔGAI(%)
*Energy*		[ΔD/DTA=3%/3 mm]	[ΔD/DTA=2%/2 mm]	[ΔD/DTA=4%/4 mm]
6 MV	Mean	0.62±1.67	−2.17±6.79	0.31±0.72
	95% confidence level.	[0.29, 0.94]	[‐3.50, −0.84]	[0.29, 0.94]
20 MV	Mean	−0.21±2.94	−0.46±6.85	−0.03±1.59
	95% confidence level.	[‐0.79, 0.38]	[‐1.80, 0.88]	[‐0.34, 0.28]

We evaluated Epiqa's correction of the linac daily outputs to analyze the result of this calibration factor. In general, Epiqa data evaluation requires an EPID image of a $10\times 10\;{\text{cm}}^{2}$ field with 50 MU acquired during the same measurement session. In order to evaluate a clinical field without taking this factor into account, the daily field was replaced by the calibration day one. The difference in term of GAI was evaluated for each clinical field and both $10\times 10\;{\text{cm}}^{2}$ fields.

**Figure 6:**
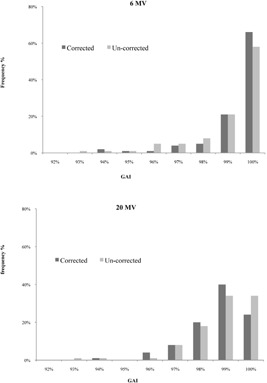
Results of Epiqa with and without linac monitor daily variation, 6 and 20 MV photon energies (ΔD/DTA=3%/3 mm).

**Figure 7:**
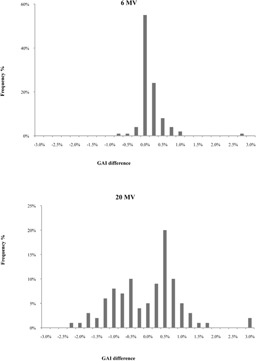
Comparison of Epiqa linac daily outputs (corrected‐uncorrected), 6 and 20 MV photon energies (ΔD=3%,DTA=3 mm).

Table 3 summarizes the deviation between corrected and noncorrected data from linac daily outputs. Output data show a mean deviation of 0.1%±0.6% and −0.2%±1.4%, respectively, for 6 MV and 20 MV photon energies. The mean gamma criteria values for corrected data were 99.5%±0.9% and 97.9%±1.7%, respectively, for 6 MV and 20 MV photon energies (Fig. 6). Since VPD does not permit this correction, we used for Epiqa an acquired $10\times 10\;{\text{cm}}^{2}$ reference field from the calibration data in order to ignore this factor. Nevertheless, the linac daily output's effect was assessed. The related results show a slight improvement for the low energy (Fig. 7). Statistical analysis did not display a significant difference (p>0.05) for either energy.

**Table 3:** Summary of Epiqa results, deviation between two methods of analysis (calibration image or daily image).

		ΔGAI(%)	ΔGAI(%)	ΔGAI(%)
*Energy*		[ΔD/DTA=3%/3 mm]	[ΔD/DTA=2%/2 mm]	[ΔD/DTA=4%/4 mm]
6 MV	Mean	0.07±0.57	0.38±1.56	−0.02±0.10
	95% confidence level.	[0.00, 0.14]	[0.07, 0.68]	[0.00, 0.04]
20 MV	Mean	−0.16±1.38	0.54±2.55	0.02±1.02
	95% confidence level.	[‐0.44, 0.11]	[0.04, 1.04]	[‐0.18, 0.22]

**Figure 8:**
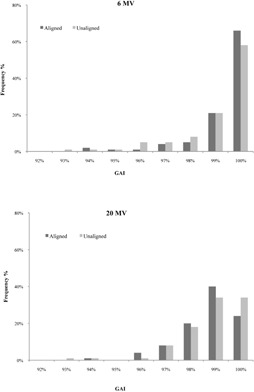
Comparison of VPD aligned vs. unaligned matrix, 6 and 20 MV photon energies (ΔD=3%,DTA=3 mm).

**Figure 9:**
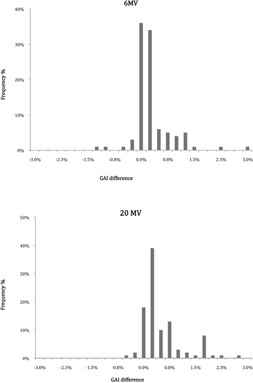
Deviation of VPD matrix (aligned‐unaligned), 6 and 20 MV photon energies (ΔD=3%,DTA=3 mm).

The evaluation of the sag effect using VPD algorithm and gamma criteria is presented in Fig. 8. The gamma parameters were 3%/3 mm. For 6 MV corrected data, the mean gamma value was 99.06%±1.3% and 98.73%±2.03% for 20 MV. The deviation between corrected and uncorrected images is shown in Fig. 9 and was 0.25%±0.69% for 6 MV and 0.40%±0.57% for 20 MV.

## IV. DISCUSSION

An intrinsic evaluation of the methods was performed using static data. We evaluated the linear response of the portal imager according to MU. The field size and dose rate dependence were also analyzed by comparing calculations and measurements.

This difference was expected since the linearity response of the detector was assessed with the GLAaS algorithm.^(7)^

For field size and dose rate parameters, the measured difference was less than 1% and 0.4% for both energies and do not impact dosimetric results in both cases.

EPID pretreatment verification is a part of our patient‐specific QA program for advanced techniques, such as IMRT or VMAT. The VPD is a fast procedure. Epiqa needs a few more minutes due to export procedures and additional image needed to automatically account for daily linac output variations.

The difference between the two software programs was much less than 1%, which translates into a good agreement between the performance of the devices and their response for the energies. For the gamma criteria, more than 99% and 98% for Epiqa and VPD, respectively, out of the 100 treatments plans analyzed fulfill the gamma criteria confidence level of 95%. For the high energy, 95% and 96% fulfill the same confidence level for Epiqa and VPD, respectively. Deviation between Epiqa and VPD did not show a significant difference (p>0.05) for the ΔD/DTA criteria of 3%/3 mm.

During pretreatment QA, the EPID and gantry are both affected by gravity during rotation due to their structural features.^(15)^ Indeed, that can lead to geometric uncertainties and can affect dosimetry results, including gamma criteria evaluation. While sag effect is already present during treatments, it's not taken into account in the calculation during the treatment planning procedure. We took advantage of the realignment tool integrated in VPD to compare the impact the gamma criteria evaluation for clinical data. Results showed a slight enhancement of the gamma criteria value for aligned data compared to raw VPD data.

## V. CONCLUSIONS

For pretreatment QA, both VPD and Epiqa software showed similar results for clinical data and for low and high energies. Epiqa gives a better linearity response for low doses and a dedicated tool to analyze static fields. VPD is a system integrated into Eclipse; it is faster and more convenient to use for clinical routine than Epiqa. Although the latter needs to export images and treatment plan from the record and verify system in order to be analyzed, it is a reliable and independent system. The GLAaS algorithm, used by Epiqa, checks the TPS dose calculation, while VPD algorithm controls only the fluence and is also a dose calculation instrument. In our opinion, Epiqa could be an alternative to VPD for routine use and a reference to audit the TPS.

## ACKNOWLEDGMENTS

The authors would like to thank EPIdos for valuable discussions. This work was supported by Mr. Dinet, Chief Executive Officer of S.A. Hartmann and the authors gratefully give him acknowledgments. All the people who reviewed the manuscript are thanked.
